# Supplementary material for: Structural basis of a public antibody response to SARS-CoV-2
Source: bioRxiv. 2020 Jun 9:2020.06.08.141267. Preprint. [Version 1] doi: 10.1101/2020.06.08.141267 (PMC7302194; doi:10.1101/2020.06.08.141267)
Supplement: 1 [file NIHPP2020.06.08.141267-supplement-1.pdf]

bioRxiv preprint doi: https://doi.org/10.1101/2020.06.08.141267. this version posted June 9, 2020. The copyright holder for this preprint (which was not certified by peer review) is the author/funder. It is made available under a CC-BY 4.0 International license.

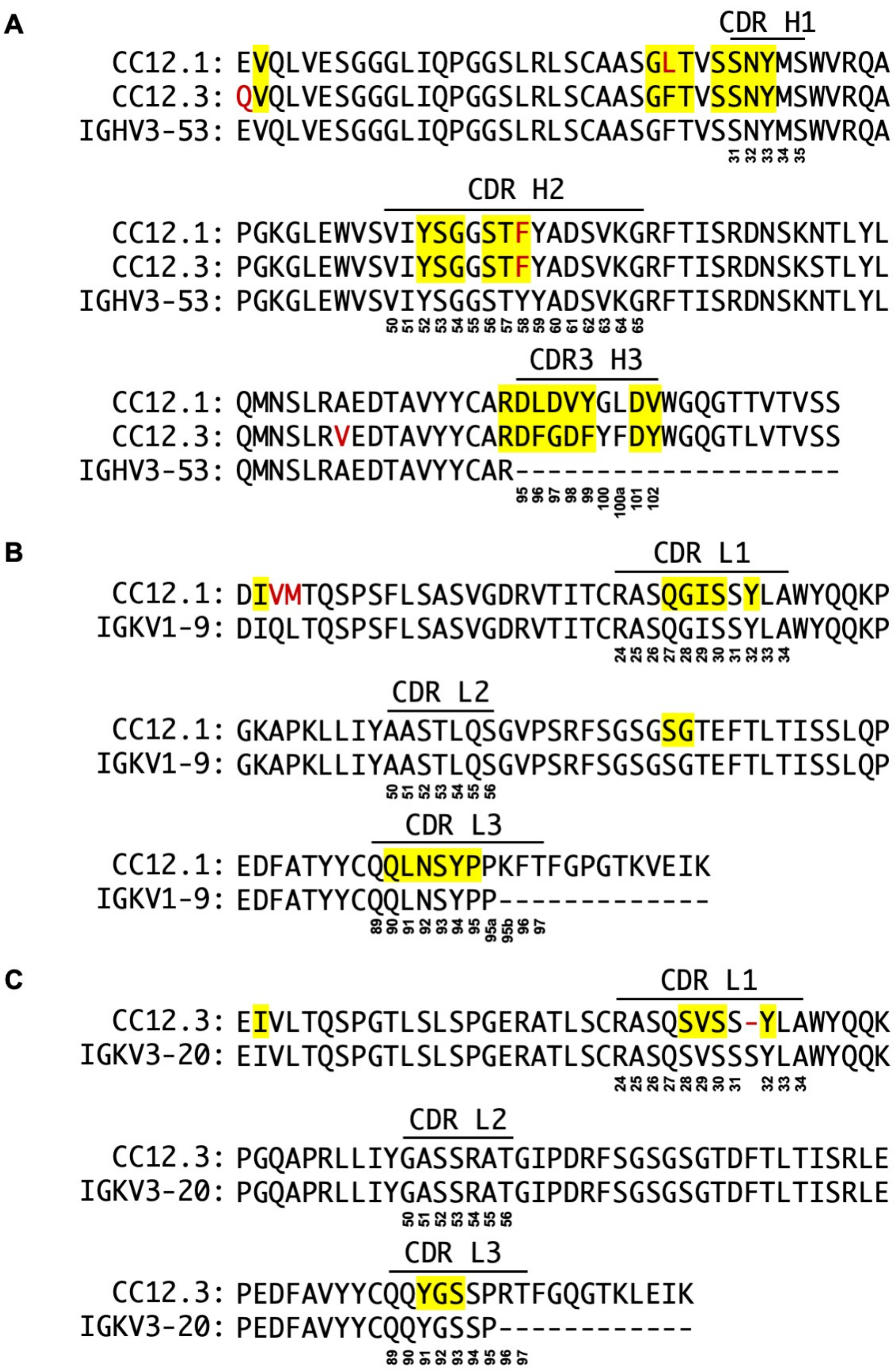

Fig. S1. Comparison of CC12.1 and CC12.3 sequence to the IGHV3-53 germline sequence. (A) Alignment of the heavy chain variable domain sequences of CC12.1 and

bioRxiv preprint doi: https://doi.org/10.1101/2020.06.08.141267. this version posted June 9, 2020. The copyright holder for this preprint (which was not certified by peer review) is the author/funder. It is made available under a CC-BY 4.0 International license.

CC12.3 with the germline IGHV3-53 sequence **(B)** Alignment of the light-chain variable domain sequence of CC12.1 with the germline IGKV1-9 sequence. **(C)** Alignment of the light-chain variable domain sequence of CC12.3 with the germline IGKV3-20 sequence. The regions that correspond to CDR H1, H2, H3, L1, L2, and L3 are indicated. Residues that differ from the germline are highlighted in red. Residue positions in the CDRs are labeled according to the Kabat numbering scheme. Residues that interact with the RBD are highlighted in yellow.

bioRxiv preprint doi: https://doi.org/10.1101/2020.06.08.141267. this version posted June 9, 2020. The copyright holder for this preprint (which was not certified by peer review) is the author/funder. It is made available under a CC-BY 4.0 International license.

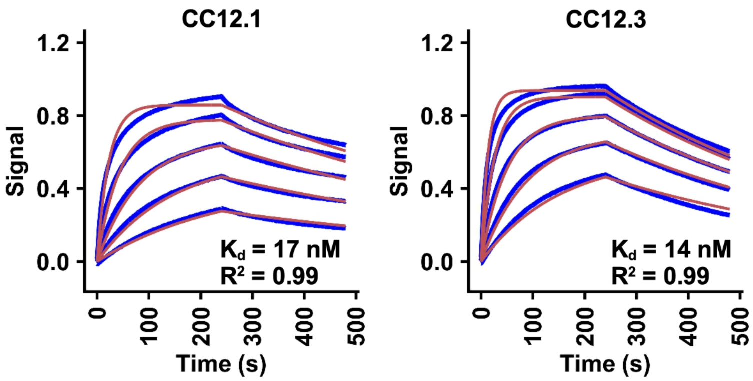

Fig. S2. Sensorgrams for binding of CC12.1 and CC12.3 Fabs to SARS-CoV-2 RBD. Binding kinetics of CC12.1 and CC12.3 Fab against SARS-CoV-2 RBD were measured by biolayer interferometry (BLI). Y-axis represents the response. Blue lines represent the response curves and red lines represent the 1:1 binding model. Binding kinetics were measured for five concentrations of Fab at 2-fold dilution ranging from 500 nM to 31.25 nM. The K_d and R^2 of the fitting are indicated.

bioRxiv preprint doi: https://doi.org/10.1101/2020.06.08.141267. this version posted June 9, 2020. The copyright holder for this preprint (which was not certified by peer review) is the author/funder. It is made available under a CC-BY 4.0 International license.

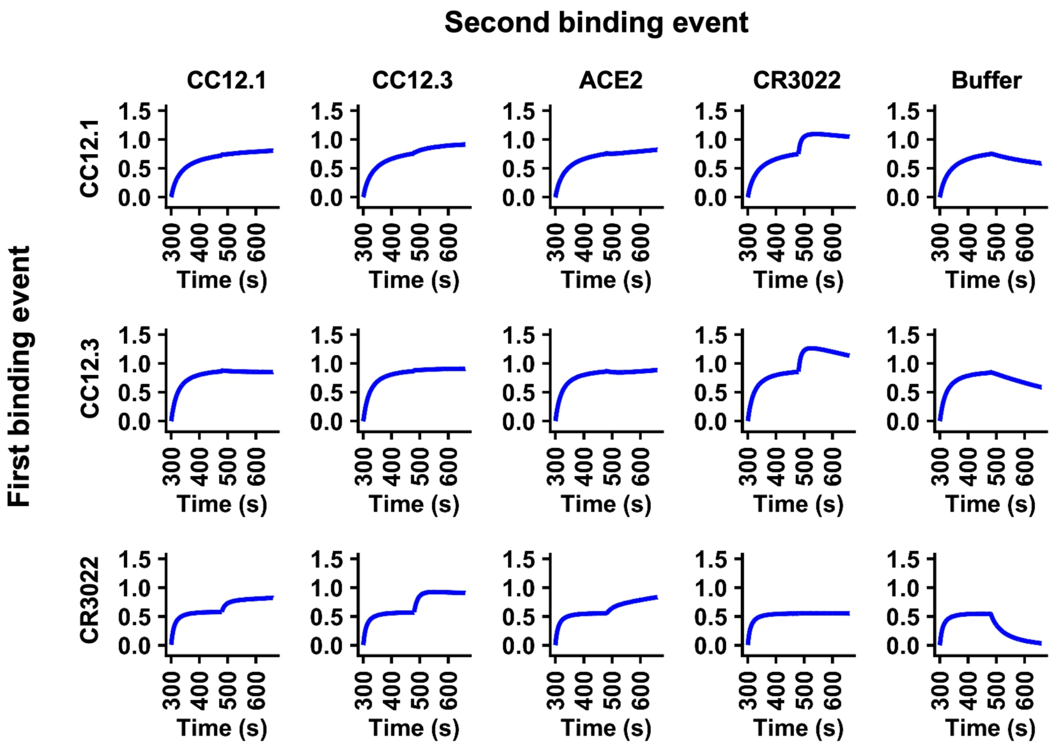

Fig. S3. Competition assay between different Fabs and ACE2. Competition between CC12.1, CC12.3, CR3022, and ACE2 was measured by biolayer interferometry (BLI). Y-axis represents the response. The biosensor was first loaded with SARS-CoV-2 RBD, followed by two binding events: 1) CC12.1, CC12.3, or CR3022, and 2) CC12.1, CC12.3, or CR3022, ACE2, and buffer (negative control). A period of 180 s was used for each of the binding events. A further increase in signal during the second binding event (starting at 480 s time point) indicates lack of competition with the first ligand.

bioRxiv preprint doi: https://doi.org/10.1101/2020.06.08.141267. this version posted June 9, 2020. The copyright holder for this preprint (which was not certified by peer review) is the author/funder. It is made available under a CC-BY 4.0 International license.

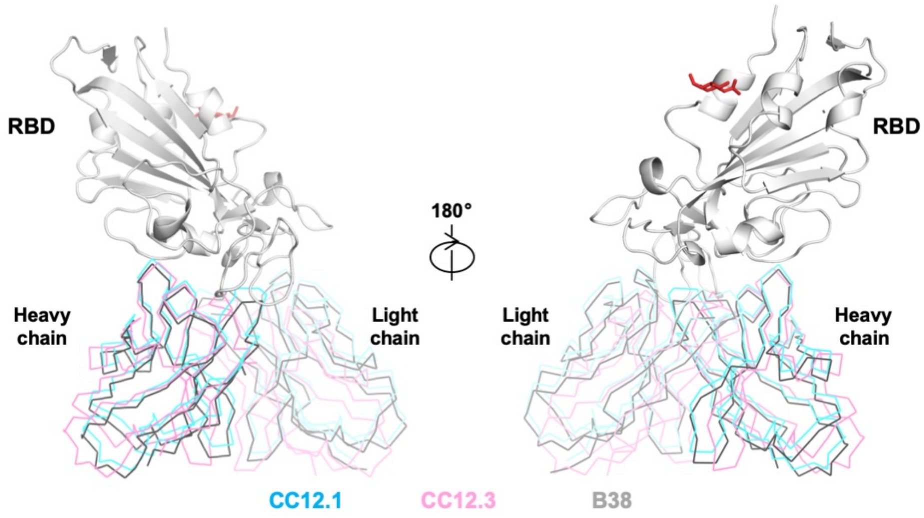

483

484 **Fig. S4. Structural comparison of the binding modes among IGHV3-53 antibodies.**

485 The binding modes of CC12.1 (cyan), CC12.3 (pink), and B38 (gray) to SARS-CoV-2

486 (white) are compared. B38 in complex with SARS-CoV-2 RBD was from PDB 7BZ5 (*23*).

487 The N-glycan observed at SARS-CoV-2 RBD N343 is shown in red.

bioRxiv preprint doi: https://doi.org/10.1101/2020.06.08.141267. this version posted June 9, 2020. The copyright holder for this preprint (which was not certified by peer review) is the author/funder. It is made available under a CC-BY 4.0 International license.

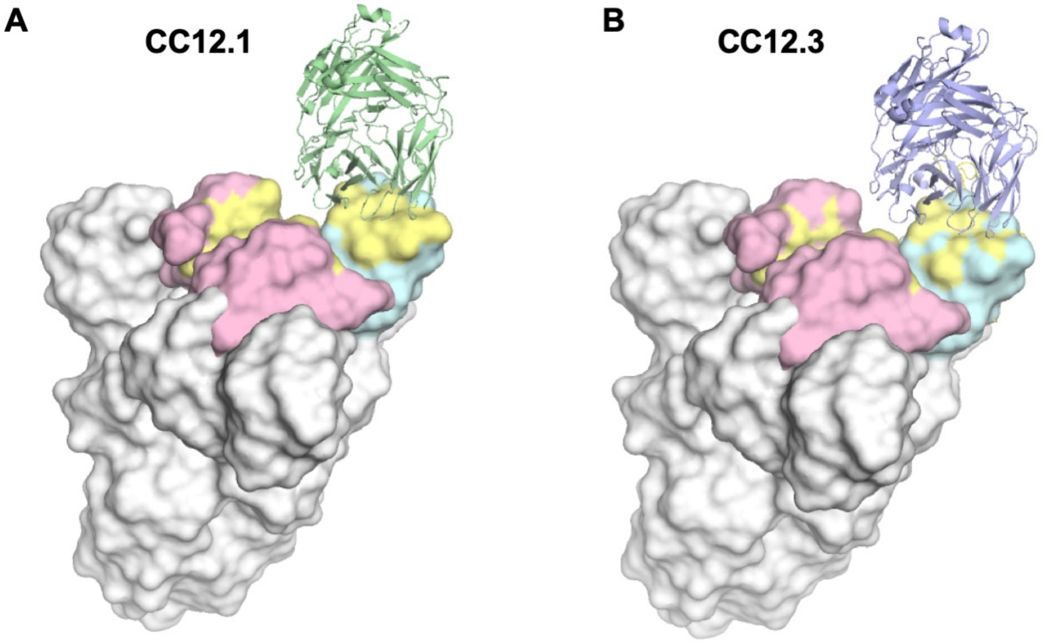

Fig. S5. Modelling the binding of CC12.1 and CC12.3 on the homotrimeric spike (S) protein. The S trimer is shown with one RBD in the up conformation (cyan) and two RBDs in the down conformation (pink). The CC12.1 and CC12.3 epitopes are shown in yellow. **(A)** Model of the binding of CC12.1 (green) to the RBD up conformation. **(B)** Model of the binding of CC12.3 (blue). PDB 6VSB is used in the modeling (*48*). The complete epitopes of CC12.1 and CC12.3 are accessible only when the RBD is in the up, but not the down, conformation.

bioRxiv preprint doi: https://doi.org/10.1101/2020.06.08.141267. this version posted June 9, 2020. The copyright holder for this preprint (which was not certified by peer review) is the author/funder. It is made available under a CC-BY 4.0 International license.

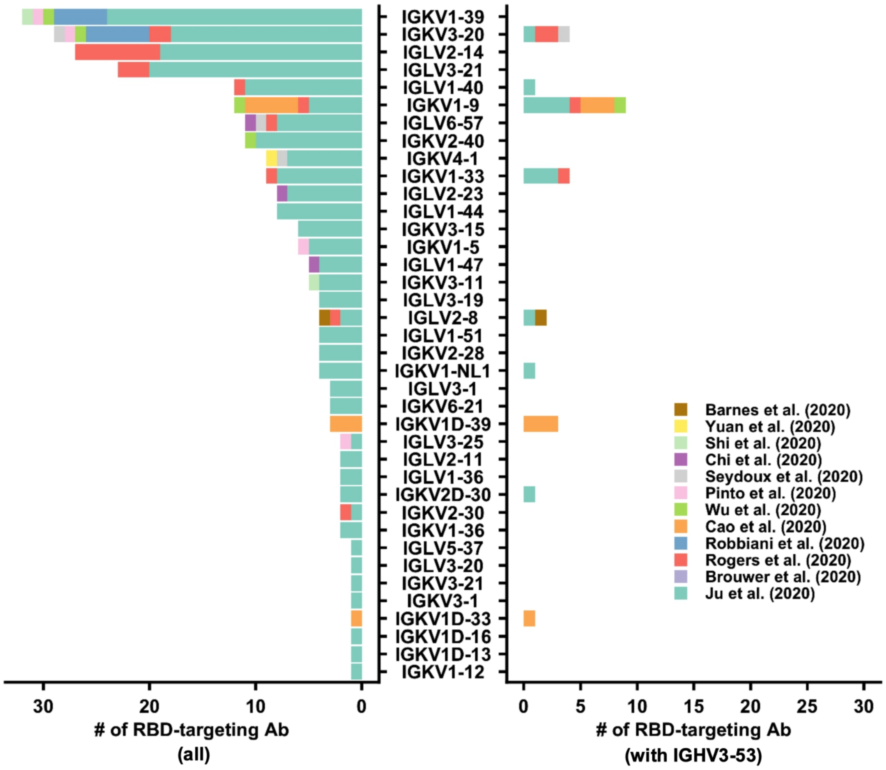

Fig. S6. Light-chain germline gene use in SARS-CoV-2 RBD-targeting antibodies. The distribution of light-chain germline gene use of SARS-CoV-2 RBD-targeting antibodies that have been recently isolated (*17-28*) is shown on the left. The distribution of light-chain germline gene use in antibodies that target SARS-CoV-2 RBD to the subset of antibodies that pair with IGHV3-53 is shown on the right.

bioRxiv preprint doi: https://doi.org/10.1101/2020.06.08.141267. this version posted June 9, 2020. The copyright holder for this preprint (which was not certified by peer review) is the author/funder. It is made available under a CC-BY 4.0 International license.

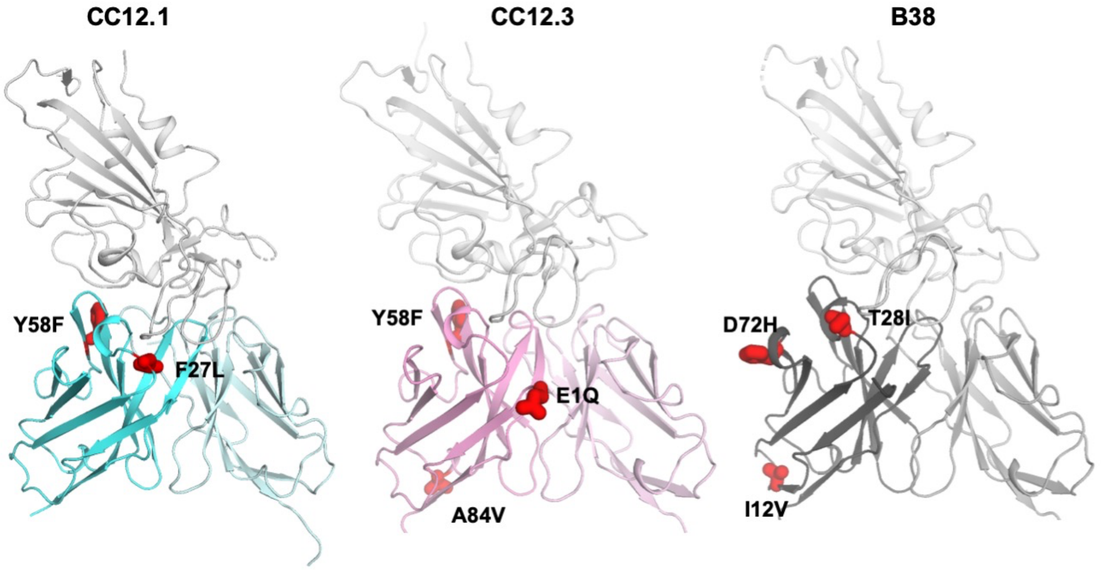

Fig. S7. Locations of heavy chain somatic mutations. Somatic mutations on the heavy chains of CC12.1, CC12.3, and B38 are labeled and shown in red on the structure. Somatic mutations contribute minimally to the antibody binding interactions.

bioRxiv preprint doi: https://doi.org/10.1101/2020.06.08.141267. this version posted June 9, 2020. The copyright holder for this preprint (which was not certified by peer review) is the author/funder. It is made available under a CC-BY 4.0 International license.

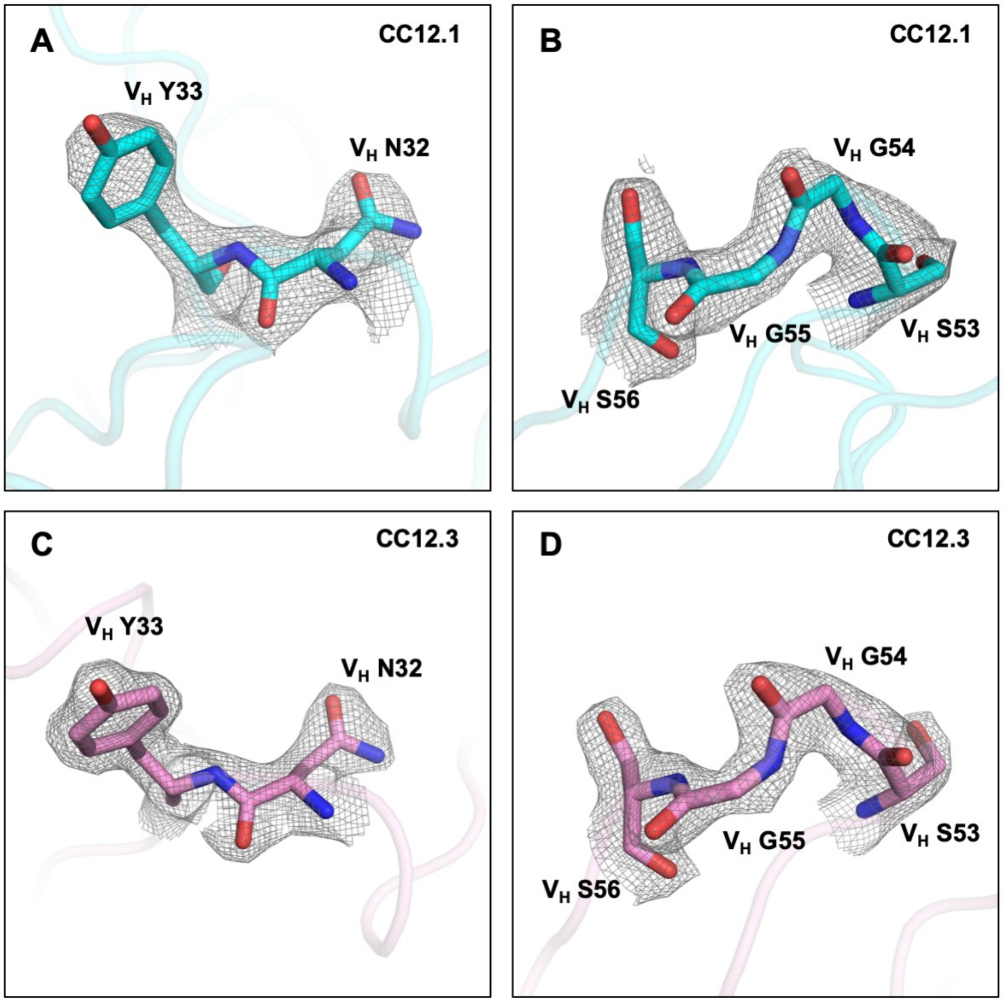

Fig. S8. Electron density maps for IGHV3-53-encoded paratope regions of CC12.1 and CC12.3. (A-B) Final 2Fo-Fc electron density maps for the IGHV3-53-encoded paratope regions around V_H N32 and Y33 (CDR H1) and V_H S53 to S56 (CDR H2) of CC12.1, both contoured at 1.2 σ. **(C-D)** Final 2Fo-Fc electron density maps for IGHV3-53-encoded paratope regions around V_H N32 and Y33 and V_H S53 to S56 of CC12.3, both contoured at 1.8 σ.

bioRxiv preprint doi: https://doi.org/10.1101/2020.06.08.141267. this version posted June 9, 2020. The copyright holder for this preprint (which was not certified by peer review) is the author/funder. It is made available under a CC-BY 4.0 International license.

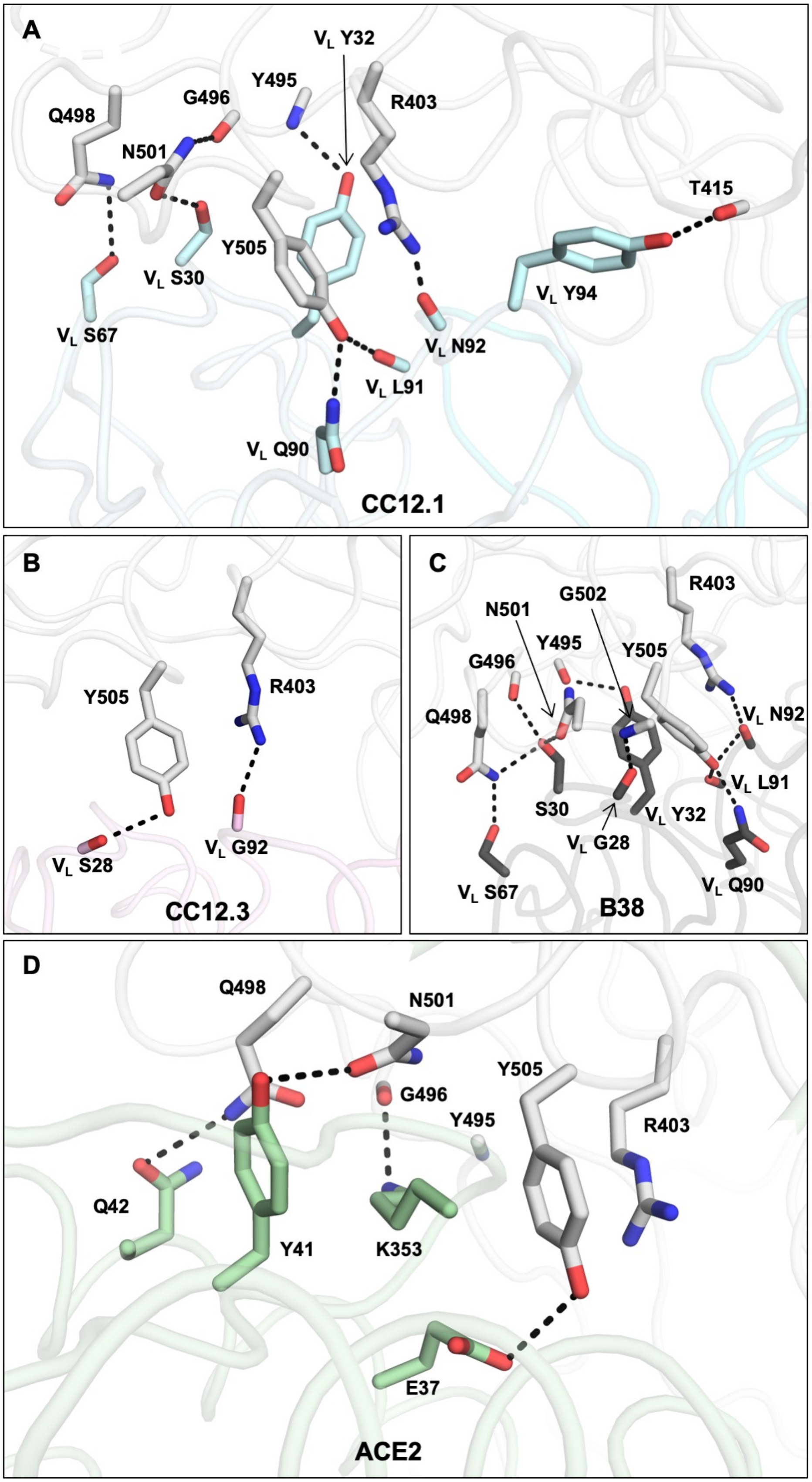

515

bioRxiv preprint doi: https://doi.org/10.1101/2020.06.08.141267. this version posted June 9, 2020. The copyright holder for this preprint (which was not certified by peer review) is the author/funder. It is made available under a CC-BY 4.0 International license.

Fig. S9. Interactions between the light chain and the RBD. (A-C) Representative interactions between SARS-CoV-2 RBD and the light chain in **(A)** CC12.1, **(B)** CC12.3, and **(C)** B38 (PDB 7BZ5) (*23*) are shown. RBD is in white. Oxygen atoms are in red. Nitrogen atoms are in blue. Hydrogen bonds are represented by dashed lines. The light chains from both CC12.1 and B38 form an extensive hydrogen bond network with the RBD, whereas the interaction between the light chain of CC12.3 and the RBD is minimal. **(D)** The interaction between ACE2 and RBD residues (PDB 6M0J) (*12*) that are shown in **(A-C)**. None of the interactions between the light chain and RBD mimic those between ACE2 and RBD.

bioRxiv preprint doi: https://doi.org/10.1101/2020.06.08.141267. this version posted June 9, 2020. The copyright holder for this preprint (which was not certified by peer review) is the author/funder. It is made available under a CC-BY 4.0 International license.

Table S1. X-ray data collection and refinement statistics

**Data collection**				
	CC12.1 + RBD	CC12.3 + RBD	CC12.1 + RBD + CR3022	CC12.3 + RBD + CR3022
Beamline	SSRL 12-1	SSRL 12-1	SSRL 12-1	SSRL 12-1
Wavelength (Å)	0.97946	0.97946	0.97946	0.97946
Space group	$P\ 1\ 2_1\ 1$	$P\ 1\ 2_1\ 1$	$P\ 4_1\ 2_1\ 2$	$P\ 4_1\ 2_1\ 2$
Unit cell parameters				
a, b, c (Å)	80.7, 143.5, 81.5	56.1, 105.6, 165.9	109.8, 109.8, 235.7	110.9, 110.9, 228.5
α, β, γ (°)	90, 118.7, 90	90, 92.8, 90	90, 90, 90	90, 90, 90
Resolution (Å) [a]	50.0-3.20 (3.27-3.20)	50.0–2.33 (2.38-2.33)	50.0-2.70 (2.76-2.70)	50-2.90 (2.97-2.90)
Unique reflections [a]	25,802 (1,234)	78,783 (7,298)	40,463 (3,945)	32,870 (3,160)
Redundancy [a]	1.5 (1.3)	2.4 (2.1)	5.7 (3.7)	13.6 (12.6)
Completeness (%) [a]	88.3 (42.5)	97.6 (98.8)	99.9 (100.0)	99.9 (100.0)
$<I/\sigma_I>$ [a]	7.4 (1.1)	10.2 (1.0)	16.2 (1.2)	20.1 (1.1)
R_{sym}[b] (%) [a]	16.3 (57.4)	14.3 (95.4)	16.7 (>100)	12.9 (>100)
R_{pim}[b] (%) [a]	11.6 (45.0)	7.0 (50.8)	5.3 (41.6)	3.6 (46.7)
$CC_{1/2}$[c] (%) [a]	96.6 (61.7)	98.7 (50.8)	100.6 (60.3)	99.6 (65.2)
**Refinement statistics**				
Resolution (Å)	39.7–3.11	41.7–2.34	41.6–2.70	42.3–2.88
Reflections (work)	25,776	78,774	40,462	32,868
Reflections (test)	1,289	3,800	1,963	1,654
R_{cryst}[d] / R_{free}[e] (%)	21.3/26.7	18.4/21.9	17.7/22.4	21.9 (25.9)
No. of atoms	9,608	10,309	8,346	8,191
Macromolecules	9,580	9,632	8148	8,152
Glycans	28	28	28	39
Solvent	0	649	170	0
Average *B*-value (Å^2)	70	39	49	76
Macromolecules	70	39	49	76
Glycans	98	65	72	94
Solvent	-	41	43	-
Wilson *B*-value (Å^2)	70	35	53	80
**RMSD from ideal geometry**				
Bond length (Å)	0.003	0.004	0.003	0.003
Bond angle (°)	0.68	0.78	0.70	0.78
**Ramachandran statistics (%)**				
Favored	94.7	96.5	96.8	95.9
Outliers	0.16	0.24	0.10	0.19
**PDB code**	6XC2	6XC4	6XC3	6XC7

[a] Numbers in parentheses refer to the highest resolution shell.
[b] $R_{sym} = \Sigma_{hkl} \Sigma_i | I_{hkl,i} - <I_{hkl}> | / \Sigma_{hkl} \Sigma_i I_{hkl,i}$ and $R_{pim} = \Sigma_{hkl} (1/(n-1))^{1/2} \Sigma_i | I_{hkl,i} - <I_{hkl}> | / \Sigma_{hkl} \Sigma_i I_{hkl,i}$, where $I_{hkl,i}$ is the scaled intensity of the ith measurement of reflection h, k, l, $<I_{hkl}>$ is the average intensity for that reflection, and *n* is the redundancy.
[c] $CC_{1/2}$ = Pearson correlation coefficient between two random half datasets.
[d] $R_{cryst} = \Sigma_{hkl} | F_o - F_c | / \Sigma_{hkl} | F_o |$ x 100, where F_o and F_c are the observed and calculated structure factors, respectively.
[e] R_{free} was calculated as for R_{cryst}, but on a test set comprising 5% of the data excluded from refinement.

bioRxiv preprint doi: https://doi.org/10.1101/2020.06.08.141267. this version posted June 9, 2020. The copyright holder for this preprint (which was not certified by peer review) is the author/funder. It is made available under a CC-BY 4.0 International license.

534 **Table S2. A list of previously reported SARS-CoV-2 RBD-targeting antibodies.**

Antibody name	Heavy chain	Light chain	CDR H3 length	Reference
S124	IGHV2-26	IGKV1-39	15	Pinto et al. (2020)
S309	IGHV1-18	IGKV3-20	18	Pinto et al. (2020)
S315	IGHV3-7	IGLV3-25	15	Pinto et al. (2020)
S303	IGHV3-23	IGKV1-5	15	Pinto et al. (2020)
P1A-1C7	IGHV1-46	IGKV1-39	13	Ju et al. (2020)
P1A-1C10	IGHV1-69	IGKV1-5	14	Ju et al. (2020)
P1A-1C11	IGHV1-69	IGKV1-5	14	Ju et al. (2020)
P1A-1C6	IGHV3-13	IGKV1-39	17	Ju et al. (2020)
P1A-1D3	IGHV3-13	IGKV1-39	16	Ju et al. (2020)
P1A-1C2	IGHV3-23	IGKV1-36	8	Ju et al. (2020)
P1A-1B2	IGHV3-30	IGLV2-14	10	Ju et al. (2020)
P1A-1C1	IGHV3-33	IGKV1D-13	15	Ju et al. (2020)
P1A-1D1	IGHV3-53	IGLV2-8	10	Ju et al. (2020)
P1A-1D5	IGHV3-53	IGKV1-33	13	Ju et al. (2020)
P1A-1D6	IGHV3-53	IGKV1-33	13	Ju et al. (2020)
P2A-1A10	IGHV1-2	IGKV2-40	17	Ju et al. (2020)
P2B-1A4	IGHV1-2	IGKV2-40	17	Ju et al. (2020)
P2B-1B2	IGHV1-2	IGKV2-40	17	Ju et al. (2020)
P2B-2G1	IGHV1-2	IGKV2-40	17	Ju et al. (2020)
P2B-2G12	IGHV1-2	IGKV2-40	17	Ju et al. (2020)
P2C-1A10	IGHV1-2	IGKV2-40	17	Ju et al. (2020)
P2C-1B10	IGHV1-2	IGKV2-40	17	Ju et al. (2020)
P2C-1D6	IGHV1-2	IGKV2-40	17	Ju et al. (2020)
P2C-1D12	IGHV1-2	IGKV2-40	17	Ju et al. (2020)
P2C-1F10	IGHV1-2	IGKV2-40	17	Ju et al. (2020)
P2B-1F8	IGHV1-2	IGKV3-20	12	Ju et al. (2020)
P2B-2G9	IGHV1-2	IGKV3-20	12	Ju et al. (2020)
P2B-1C3	IGHV1-46	IGKV1-5	13	Ju et al. (2020)
P2C-1C10	IGHV1-69	IGKV3-11	9	Ju et al. (2020)
P2B-2G10	IGHV1-69	IGKV1-39	9	Ju et al. (2020)
P2B-1F11	IGHV1-69	IGLV1-40	15	Ju et al. (2020)
P2B-1D9	IGHV2-5	IGLV1-47	14	Ju et al. (2020)
P2B-1E2	IGHV2-5	IGKV1-5	10	Ju et al. (2020)
P2B-1E4	IGHV2-5	IGLV2-14	9	Ju et al. (2020)
P2B-1F4	IGHV2-70	IGLV1-44	12	Ju et al. (2020)
P2C-1A3	IGHV3-11	IGKV1-9	10	Ju et al. (2020)
P2B-1D6	IGHV3-15	IGLV1-44	22	Ju et al. (2020)
P2C-1B12	IGHV3-15	IGLV6-57	11	Ju et al. (2020)
P2B-1F9	IGHV3-15	IGKV1-NL1	14	Ju et al. (2020)
P2C-1D5	IGHV3-23	IGLV3-21	12	Ju et al. (2020)
P2B-1B4	IGHV3-30	IGKV1-39	20	Ju et al. (2020)
P2B-1F2	IGHV3-33	IGLV2-11	9	Ju et al. (2020)
P2B-2G4	IGHV3-33	IGLV2-11	9	Ju et al. (2020)
P2C-1C8	IGHV3-33	IGKV2D-30	11	Ju et al. (2020)
P2A-1B3	IGHV3-48	IGKV3-20	14	Ju et al. (2020)
P2B-1B11	IGHV3-48	IGKV3-20	14	Ju et al. (2020)
P2B-1B12	IGHV3-48	IGKV3-20	14	Ju et al. (2020)
P2B-1C4	IGHV3-48	IGKV3-20	14	Ju et al. (2020)
P2B-1E11	IGHV3-48	IGKV3-20	14	Ju et al. (2020)
P2B-2H7	IGHV3-48	IGKV3-20	14	Ju et al. (2020)

bioRxiv preprint doi: https://doi.org/10.1101/2020.06.08.141267. this version posted June 9, 2020. The copyright holder for this preprint (which was not certified by peer review) is the author/funder. It is made available under a CC-BY 4.0 International license.

P2B-1G12	IGHV3-48	IGKV3-20	14	Ju et al. (2020)
P2C-1E5	IGHV3-48	IGKV3-20	14	Ju et al. (2020)
P2B-1A10	IGHV3-53	IGKV1-33	13	Ju et al. (2020)
P2B-1F5	IGHV3-53	IGKV1-NL1	12	Ju et al. (2020)
P2C-1D7	IGHV3-53	IGKV2D-30	10	Ju et al. (2020)
P2B-1G1	IGHV3-66	IGKV3-20	9	Ju et al. (2020)
P2C-1E1	IGHV3-66	IGKV3-11	7	Ju et al. (2020)
P2C-1F11	IGHV3-66	IGKV3-20	9	Ju et al. (2020)
P2A-1A8	IGHV3-9	IGLV2-14	21	Ju et al. (2020)
P2B-1B10	IGHV3-9	IGLV2-14	21	Ju et al. (2020)
P2B-1C10	IGHV3-9	IGLV2-14	21	Ju et al. (2020)
P2B-1D3	IGHV3-9	IGLV2-14	21	Ju et al. (2020)
P2B-2H4	IGHV3-9	IGLV2-14	21	Ju et al. (2020)
P2C-1A5	IGHV3-9	IGLV2-14	21	Ju et al. (2020)
P2C-1A8	IGHV3-9	IGLV2-14	21	Ju et al. (2020)
P2C-1B1	IGHV3-9	IGLV2-14	21	Ju et al. (2020)
P2C-1C12	IGHV3-9	IGLV2-14	21	Ju et al. (2020)
P2C-1A6	IGHV3-9	IGLV2-14	21	Ju et al. (2020)
P2A-1A9	IGHV3-9	IGLV1-40	15	Ju et al. (2020)
P2C-1A1	IGHV3-9	IGLV1-40	15	Ju et al. (2020)
P2B-2G11	IGHV3-9	IGLV1-40	15	Ju et al. (2020)
P2B-1E12	IGHV3-9	IGLV3-20	15	Ju et al. (2020)
P2B-2F6	IGHV4-38	IGLV2-8	18	Ju et al. (2020)
P2A-1B10	IGHV4-39	IGLV1-47	18	Ju et al. (2020)
P2B-1B9	IGHV4-39	IGKV1-NL1	7	Ju et al. (2020)
P2B-2F11	IGHV4-39	IGKV1-NL1	7	Ju et al. (2020)
P2B-1G8	IGHV4-39	IGKV1-5	9	Ju et al. (2020)
P2B-1A1	IGHV4-59	IGLV2-14	12	Ju et al. (2020)
P2B-1D11	IGHV4-59	IGLV3-25	20	Ju et al. (2020)
P2B-1F11	IGHV4-59	IGKV1-39	13	Ju et al. (2020)
P2C-1A7	IGHV5-51	IGLV3-1	15	Ju et al. (2020)
P2B-1A12	IGHV7-4-1	IGKV1-39	14	Ju et al. (2020)
P2B-1G5	IGHV7-4-1	IGLV3-21	10	Ju et al. (2020)
P3A-1F1	IGHV3-13	IGKV1-39	15	Ju et al. (2020)
P3A-1G8	IGHV3-64	IGLV1-44	17	Ju et al. (2020)
P4A-2A10	IGHV1-46	IGLV1-40	24	Ju et al. (2020)
P4B-1F6	IGHV1-69	IGLV2-23	13	Ju et al. (2020)
P4B-1E11	IGHV2-5	IGLV1-36	16	Ju et al. (2020)
P4A-2A2	IGHV3-23	IGLV1-51	12	Ju et al. (2020)
P4A-2A8	IGHV3-23	IGLV3-21	9	Ju et al. (2020)
P4A-2C1	IGHV3-23	IGKV2-28	14	Ju et al. (2020)
P4A-1H5	IGHV3-30	IGKV1-39	19	Ju et al. (2020)
P4B-1G2	IGHV3-30	IGKV1-39	19	Ju et al. (2020)
P4A-2B3	IGHV3-30	IGKV1-39	19	Ju et al. (2020)
P4A-1H6	IGHV3-30	IGKV1-39	19	Ju et al. (2020)
P4B-1G5	IGHV3-30	IGLV3-21	20	Ju et al. (2020)
P4A-2E10	IGHV3-30	IGKV1-39	19	Ju et al. (2020)
P4B-1E3	IGHV3-30	IGKV1-39	19	Ju et al. (2020)
P4A-2D9	IGHV3-30	IGKV1-39	19	Ju et al. (2020)
P4B-1F4	IGHV3-30	IGKV2-30	20	Ju et al. (2020)
P4B-1E7	IGHV3-43D	IGKV3-1	18	Ju et al. (2020)
P4B-1F10	IGHV3-7	IGKV3-21	11	Ju et al. (2020)
P4A-2D1	IGHV3-9	IGKV1-12	11	Ju et al. (2020)

bioRxiv preprint doi: https://doi.org/10.1101/2020.06.08.141267. this version posted June 9, 2020. The copyright holder for this preprint (which was not certified by peer review) is the author/funder. It is made available under a CC-BY 4.0 International license.

P4A-2D2	IGHV4-39	IGKV3-20	14	Ju et al. (2020)
P4B-1E12	IGHV4-59	IGLV1-44	9	Ju et al. (2020)
P4A-2C12	IGHV5-51	IGLV1-44	13	Ju et al. (2020)
P8A-1A8	IGHV3-23	IGLV3-21	9	Ju et al. (2020)
P8A-1C6	IGHV3-30	IGKV1-33	18	Ju et al. (2020)
P8A-1A5	IGHV5-51	IGLV1-47	16	Ju et al. (2020)
P8A-1D5	IGHV6-1	IGKV3-20	14	Ju et al. (2020)
P5A-1A1	IGHV1-24	IGKV2-28	13	Ju et al. (2020)
P5A-1C8	IGHV1-46	IGKV1-33	20	Ju et al. (2020)
P5A-2D5	IGHV1-46	IGLV1-40	22	Ju et al. (2020)
P5A-2C8	IGHV1-46	IGLV2-23	13	Ju et al. (2020)
P5A-2E9	IGHV1-46	IGLV2-14	20	Ju et al. (2020)
P5A-3B8	IGHV1-46	IGLV2-23	14	Ju et al. (2020)
P5A-3A11	IGHV1-69	IGKV1-39	12	Ju et al. (2020)
P5A-3C10	IGHV1-69	IGLV6-57	20	Ju et al. (2020)
P5A-1A2	IGHV1-8	IGLV1-40	19	Ju et al. (2020)
P5A-1C11	IGHV1-8	IGLV3-21	15	Ju et al. (2020)
P5A-2F11	IGHV1-8	IGKV4-1	13	Ju et al. (2020)
P5A-3B9	IGHV1-8	IGKV1-36	13	Ju et al. (2020)
P5A-2C12	IGHV2-5	IGKV3-11	14	Ju et al. (2020)
P5A-3C12	IGHV2-5	IGKV4-1	17	Ju et al. (2020)
P5A-3C3	IGHV2-5	IGLV6-57	10	Ju et al. (2020)
P5A-3C1	IGHV3-11	IGLV3-21	11	Ju et al. (2020)
P5A-1C4	IGHV3-13	IGKV1-39	18	Ju et al. (2020)
P5A-2G8	IGHV3-13	IGKV1-39	11	Ju et al. (2020)
P5A-2D3	IGHV3-13	IGKV1-39	14	Ju et al. (2020)
P5A-3B10	IGHV3-13	IGKV1-39	14	Ju et al. (2020)
P5A-1D8	IGHV3-13	IGLV3-19	16	Ju et al. (2020)
P5A-2G10	IGHV3-13	IGLV3-19	16	Ju et al. (2020)
P5A-2H6	IGHV3-15	IGLV3-19	16	Ju et al. (2020)
P5A-1D6	IGHV3-23	IGLV3-21	11	Ju et al. (2020)
P5A-2E12	IGHV3-23	IGLV3-21	12	Ju et al. (2020)
P5A-3D12	IGHV3-23	IGLV1-47	22	Ju et al. (2020)
P5A-1B6	IGHV3-30	IGKV1-33	18	Ju et al. (2020)
P5A-2E6	IGHV3-30	IGKV1-33	18	Ju et al. (2020)
P5A-1B1	IGHV3-33	IGKV3-15	12	Ju et al. (2020)
P5A-1C5	IGHV3-33	IGKV3-15	12	Ju et al. (2020)
P5A-2H7	IGHV3-33	IGKV3-15	12	Ju et al. (2020)
P5A-2G9	IGHV3-33	IGLV5-37	10	Ju et al. (2020)
P5A-2G11	IGHV3-33	IGLV2-14	15	Ju et al. (2020)
P5A-1B8	IGHV3-53	IGKV1-9	7	Ju et al. (2020)
P5A-1D2	IGHV3-53	IGLV1-40	13	Ju et al. (2020)
P5A-1D1	IGHV3-53	IGKV1-9	9	Ju et al. (2020)
P5A-2C9	IGHV3-7	IGKV3-20	12	Ju et al. (2020)
P5A-2E4	IGHV3-7	IGKV3-20	12	Ju et al. (2020)
P5A-2G12	IGHV3-7	IGLV6-57	10	Ju et al. (2020)
P5A-2D12	IGHV3-7	IGKV2-28	16	Ju et al. (2020)
P5A-2F1	IGHV3-74	IGLV6-57	10	Ju et al. (2020)
P5A-1C10	IGHV3-9	IGLV3-21	12	Ju et al. (2020)
P5A-2E8	IGHV3-9	IGLV3-21	11	Ju et al. (2020)
P5A-3A2	IGHV3-9	IGLV3-21	12	Ju et al. (2020)
P5A-2D6	IGHV3-9	IGLV1-40	12	Ju et al. (2020)
P5A-1B12	IGHV3-9	IGLV1-51	15	Ju et al. (2020)

bioRxiv preprint doi: https://doi.org/10.1101/2020.06.08.141267. this version posted June 9, 2020. The copyright holder for this preprint (which was not certified by peer review) is the author/funder. It is made available under a CC-BY 4.0 International license.

P5A-3A6	IGHV3-9	IGLV2-14	25	Ju et al. (2020)
P5A-3D9	IGHV3-9	IGKV3-15	14	Ju et al. (2020)
P5A-1D10	IGHV3-11	IGLV2-14	19	Ju et al. (2020)
P5A-3A1	IGHV3-53	IGKV3-20	9	Ju et al. (2020)
P5A-3C8	IGHV3-53	IGKV1-9	9	Ju et al. (2020)
P5A-2D10	IGHV4-31	IGLV6-57	10	Ju et al. (2020)
P5A-2G5	IGHV4-31	IGLV3-21	12	Ju et al. (2020)
P5A-1A12	IGHV4-39	IGKV4-1	15	Ju et al. (2020)
P5A-2C7	IGHV4-39	IGLV2-23	14	Ju et al. (2020)
P5A-2F7	IGHV4-39	IGLV2-23	16	Ju et al. (2020)
P5A-2F9	IGHV4-39	IGLV2-23	12	Ju et al. (2020)
P5A-1A5	IGHV4-4	IGLV2-14	12	Ju et al. (2020)
P5A-1C6	IGHV4-4	IGLV1-40	20	Ju et al. (2020)
P5A-3A10	IGHV4-4	IGKV1-39	19	Ju et al. (2020)
P5A-1B9	IGHV4-59	IGKV4-1	20	Ju et al. (2020)
P5A-3A7	IGHV4-59	IGKV4-1	20	Ju et al. (2020)
P5A-3B1	IGHV4-59	IGKV4-1	20	Ju et al. (2020)
P5A-3B6	IGHV4-59	IGKV4-1	20	Ju et al. (2020)
P5A-2C10	IGHV4-59	IGLV3-21	15	Ju et al. (2020)
P5A-2E5	IGHV4-59	IGLV6-57	10	Ju et al. (2020)
P5A-2G4	IGHV4-59	IGKV1D-16	10	Ju et al. (2020)
P5A-2G7	IGHV4-61	IGLV2-14	18	Ju et al. (2020)
P5A-1B10	IGHV5-51	IGKV2-28	10	Ju et al. (2020)
P5A-1C9	IGHV5-51	IGLV3-19	9	Ju et al. (2020)
P5A-2D11	IGHV5-51	IGLV1-44	11	Ju et al. (2020)
P5A-3B4	IGHV5-51	IGLV1-44	11	Ju et al. (2020)
P5A-2H3	IGHV5-51	IGLV1-44	11	Ju et al. (2020)
P5A-2E1	IGHV5-51	IGLV3-21	10	Ju et al. (2020)
P5A-1B11	IGHV7-4-1	IGKV1-39	18	Ju et al. (2020)
P5A-2D7	IGHV7-4-1	IGKV6-21	8	Ju et al. (2020)
P5A-3C9	IGHV7-4-1	IGKV6-21	8	Ju et al. (2020)
P5A-3D11	IGHV7-4-1	IGKV6-21	8	Ju et al. (2020)
P16A-1A3	IGHV1-3	IGLV6-57	9	Ju et al. (2020)
P16A-1A8	IGHV1-46	IGLV3-21	18	Ju et al. (2020)
P16A-1B5	IGHV1-46	IGLV3-21	11	Ju et al. (2020)
P16A-1C6	IGHV1-46	IGLV3-21	14	Ju et al. (2020)
P16A-1C1	IGHV3-13	IGKV1-39	19	Ju et al. (2020)
P16A-1A5	IGHV3-33	IGKV1-33	13	Ju et al. (2020)
P16A-1A12	IGHV3-33	IGLV1-51	17	Ju et al. (2020)
P16A-1B1	IGHV3-74	IGLV1-36	13	Ju et al. (2020)
P16A-1B3	IGHV3-9	IGLV3-1	22	Ju et al. (2020)
P16A-1B12	IGHV4-34	IGLV1-51	14	Ju et al. (2020)
P16A-1B8	IGHV5-51	IGLV3-1	17	Ju et al. (2020)
P16A-1A7	IGHV7-4-1	IGLV3-21	12	Ju et al. (2020)
P16A-1A10	IGHV7-4-1	IGLV3-21	13	Ju et al. (2020)
P22A-1E10	IGHV1-46	IGKV3-11	13	Ju et al. (2020)
P22A-1D2	IGHV1-8	IGLV1-40	19	Ju et al. (2020)
P22A-1D8	IGHV3-23	IGKV3-15	18	Ju et al. (2020)
P22A-1D7	IGHV3-33	IGKV1-39	11	Ju et al. (2020)
P22A-1D1	IGHV3-53	IGKV1-9	9	Ju et al. (2020)
P22A-1E8	IGHV3-9	IGKV3-15	14	Ju et al. (2020)
P22A-1D5	IGHV4-39	IGLV2-23	12	Ju et al. (2020)
P22A-1E6	IGHV4-59	IGKV3-20	14	Ju et al. (2020)

bioRxiv preprint doi: https://doi.org/10.1101/2020.06.08.141267. this version posted June 9, 2020. The copyright holder for this preprint (which was not certified by peer review) is the author/funder. It is made available under a CC-BY 4.0 International license.

BD-494	IGHV3-53	IGKV1-9	9	Cao et al. (2020)
BD-498	IGHV3-66	IGKV1-9	9	Cao et al. (2020)
BD-500	IGHV3-53	IGKV1D-39	9	Cao et al. (2020)
BD-503	IGHV3-53	IGKV1D-39	9	Cao et al. (2020)
BD-504	IGHV3-66	IGKV1-9	9	Cao et al. (2020)
BD-505	IGHV3-53	IGKV1D-33	9	Cao et al. (2020)
BD-506	IGHV3-53	IGKV1-9	9	Cao et al. (2020)
BD-507	IGHV3-53	IGKV1-9	9	Cao et al. (2020)
BD-508	IGHV3-53	IGKV1D-39	9	Cao et al. (2020)
CR3022	IGHV5-51	IGKV4-1	10	Yuan et al. (2020)
CC12.1	IGHV3-53	IGKV1-9	-	Rogers et al. (2020)
CC12.2	IGHV3-53	IGKV3-20	-	Rogers et al. (2020)
CC12.3	IGHV3-53	IGKV3-20	-	Rogers et al. (2020)
CC12.4	IGHV1-2	IGLV2-8	-	Rogers et al. (2020)
CC12.5	IGHV1-2	IGLV2-14	-	Rogers et al. (2020)
CC12.6	IGHV1-2	IGLV2-14	-	Rogers et al. (2020)
CC12.7	IGHV1-2	IGLV2-14	-	Rogers et al. (2020)
CC12.8	IGHV1-2	IGLV2-14	-	Rogers et al. (2020)
CC12.9	IGHV1-2	IGLV2-14	-	Rogers et al. (2020)
CC12.10	IGHV1-2	IGLV2-14	-	Rogers et al. (2020)
CC12.11	IGHV1-2	IGLV2-14	-	Rogers et al. (2020)
CC12.12	IGHV1-2	IGLV2-14	-	Rogers et al. (2020)
CC12.13	IGHV3-53	IGKV1-33	-	Rogers et al. (2020)
CC12.14	IGHV3-21	IGKV2-30	-	Rogers et al. (2020)
CC12.15	IGHV3-48	IGLV1-40	-	Rogers et al. (2020)
CC12.16	IGHV3-33	IGLV3-21	-	Rogers et al. (2020)
CC12.17	IGHV3-30	IGLV3-21	-	Rogers et al. (2020)
CC12.18	IGHV1-46	IGLV6-57	-	Rogers et al. (2020)
CC12.19	IGHV3-23	IGLV3-21	-	Rogers et al. (2020)
COVA1-07	IGHV1-69	-	13	Brouwer et al. (2020)
COVA1-08	IGHV3-30	-	12	Brouwer et al. (2020)
COVA1-10	IGHV3-66	-	19	Brouwer et al. (2020)
COVA1-12	IGHV1-2	-	13	Brouwer et al. (2020)
COVA1-16	IGHV1-46	-	20	Brouwer et al. (2020)
COVA1-18	IGHV3-66	-	10	Brouwer et al. (2020)
COVA2-01	IGHV3-13	-	12	Brouwer et al. (2020)
COVA2-02	IGHV4-39	-	13	Brouwer et al. (2020)
COVA2-04	IGHV3-53	-	10	Brouwer et al. (2020)
COVA2-05	IGHV5-51	-	18	Brouwer et al. (2020)
COVA2-07	IGHV3-53	-	7	Brouwer et al. (2020)
COVA2-11	IGHV3-21	-	17	Brouwer et al. (2020)
COVA2-13	IGHV1-69	-	10	Brouwer et al. (2020)
COVA2-15	IGHV3-23	-	20	Brouwer et al. (2020)
COVA2-16	IGHV1-69	-	14	Brouwer et al. (2020)
COVA2-17	IGHV1-69	-	11	Brouwer et al. (2020)
COVA2-20	IGHV3-53	-	15	Brouwer et al. (2020)
COVA2-23	IGHV1-2	-	18	Brouwer et al. (2020)
COVA2-24	IGHV5-10	-	18	Brouwer et al. (2020)
COVA2-27	IGHV1-8	-	14	Brouwer et al. (2020)
COVA2-29	IGHV4-30	-	18	Brouwer et al. (2020)
COVA2-31	IGHV1-2	-	16	Brouwer et al. (2020)
COVA2-32	IGHV1-69	-	13	Brouwer et al. (2020)
COVA2-36	IGHV5-51	-	14	Brouwer et al. (2020)

bioRxiv preprint doi: https://doi.org/10.1101/2020.06.08.141267. this version posted June 9, 2020. The copyright holder for this preprint (which was not certified by peer review) is the author/funder. It is made available under a CC-BY 4.0 International license.

COVA2-39	IGHV3-53	-	15	Brouwer et al. (2020)
COVA2-44	IGHV3-30	-	13	Brouwer et al. (2020)
COVA2-45	IGHV1-2	-	22	Brouwer et al. (2020)
COVA2-46	IGHV4-39	-	10	Brouwer et al. (2020)
COVA3-05	IGHV1-24	-	14	Brouwer et al. (2020)
COVA3-06	IGHV1-69	-	16	Brouwer et al. (2020)
COVA3-09	IGHV4-59	-	12	Brouwer et al. (2020)
COVA3-10	IGHV5-51	-	14	Brouwer et al. (2020)
B5	IGHV1-2	IGKV3-20	-	Wu et al. (2020)
B38	IGHV3-53	IGKV1-9	7	Wu et al. (2020)
H2	IGHV3-9	IGKV1-39	-	Wu et al. (2020)
H4	IGHV1-2	IGKV2-40	17	Wu et al. (2020)
COV21.1	IGHV1-58	IGKV3-20	14	Robbiani et al. (2020)
COV21.2	IGHV1-58	IGKV3-20	14	Robbiani et al. (2020)
COV57.1	IGHV1-58	IGKV3-20	14	Robbiani et al. (2020)
COV57.2	IGHV1-58	IGKV3-20	14	Robbiani et al. (2020)
COV107.1	IGHV1-58	IGKV3-20	14	Robbiani et al. (2020)
COV107.2	IGHV1-58	IGKV3-20	14	Robbiani et al. (2020)
COV21.3	IGHV3-30	IGKV1-39	14	Robbiani et al. (2020)
COV21.4	IGHV3-30	IGKV1-39	12	Robbiani et al. (2020)
COV72.1	IGHV3-30	IGKV1-39	14	Robbiani et al. (2020)
COV72.2	IGHV3-30	IGKV1-39	14	Robbiani et al. (2020)
COV72.3	IGHV3-30	IGKV1-39	14	Robbiani et al. (2020)
1M-1D2	IGHV3-64	IGLV1-47	18	Chi et al. (2020)
2M-10B11	IGHV3-66	IGLV6-57	10	Chi et al. (2020)
2M-4G4	IGHV1-46	IGLV2-23	23	Chi et al. (2020)
CV5	IGHV1-46	IGKV4-1	-	Seydoux et al. (2020)
CV30	IGHV3-53	IGKV3-20	-	Seydoux et al. (2020)
CV43	IGHV3-30	IGLV6-57	-	Seydoux et al. (2020)
CA1	IGHV1-18	IGKV3-11	21	Shi et al. (2020)
CB6	IGHV3-66	IGKV1-39	11	Shi et al. (2020)
C105	IGHV3-53	IGLV2-8	-	Barnes et al. (2020)

bioRxiv preprint doi: https://doi.org/10.1101/2020.06.08.141267. this version posted June 9, 2020. The copyright holder for this preprint (which was not certified by peer review) is the author/funder. It is made available under a CC-BY 4.0 International license.

Table S3. Hydrogen bonds and salt bridges identified at the antibody-RBD interface using the PISA program.

SARS-CoV-2 RBD	Distance [Å]	CC12.1
	Hydrogen bonds	
TYR 473[OH]	3.63	VH SER 53[OG]
TYR 473[OH]	2.77	VH SER 31[O]
ASP 420[OD2]	2.51	VH SER 56[OG]
TYR 421[O]	3.39	VH SER 53[N]
LEU 455[O]	2.59	VH TYR 33[OH]
ALA 475[O]	3.02	VH ASN 32[ND2]
ALA 475[O]	3.16	VH THR 28[N]
ASN 487[OD1]	3.13	VH ARG 94[NH1]
ASN 487[OD1]	3.16	VH ARG 94[NH2]
TYR 489[OH]	3.26	VH ARG 94[NH2]
GLN 493[OE1]	2.85	VH TYR 99[OH]
TYR 505[OH]	2.95	VL LEU 91[O]
ARG 403[NH2]	2.08	VL ASN 92[O]
TYR 505[OH]	3.11	VL ASN 92[O]
SER 494[O]	3.55	VL TYR 32[OH]
GLN 498[OE1]	3.61	VL SER 67[OG]
TYR 505[OH]	2.94	VL GLN 90[NE2]
TYR 453[OH]	3.29	VL ASN 92[ND2]
THR 415[O]	2.69	VL TYR 94[OH]
	Salt bridges	
LYS 417[NZ]	3.17	VH ASP 97[OD1]

SARS-CoV-2 RBD	Distance [Å]	CC12.3
	Hydrogen bonds	
ASP 420[OD2]	2.60	VH SER 56[OG]
TYR 421[OH]	3.36	VH SER 53[OG]
TYR 421[OH]	3.72	VH SER 53[N]
LEU 455[O]	2.65	VH TYR 33[OH]
ARG 457[O]	2.81	VH SER 53[OG]
ALA 475[O]	2.97	VH ASN 32[ND2]
ALA 475[O]	3.13	VH THR 28[N]
ASN 487[OD1]	2.67	VH ARG 94[NH2]
TYR 489[OH]	2.88	VH ARG 94[NH1]
TYR 489[OH]	2.71	VH ARG 94[NH2]
SER 477[N]	3.88	VH THR 28[OG1]
TYR 473[OH]	2.74	VH SER 31[O]
ARG 457[N]	3.64	VL SER 53[OG]
TYR 495[O]	3.87	VL TYR 32[OH]
TYR 505[OH]	3.88	VL SER 93[N]
TYR 505[OH]	3.21	VL SER 28[O]

bioRxiv preprint doi: https://doi.org/10.1101/2020.06.08.141267. this version posted June 9, 2020. The copyright holder for this preprint (which was not certified by peer review) is the author/funder. It is made available under a CC-BY 4.0 International license.

SUPPLEMENTARY REFERENCES

40. D. C. Ekiert *et al.*, A highly conserved neutralizing epitope on group 2 influenza A viruses. *Science* **333**, 843-850 (2011).

41. Z. Otwinowski, W. Minor, Processing of X-ray diffraction data collected in oscillation mode. *Methods Enzymol* **276**, 307-326 (1997).

42. A. J. McCoy *et al.*, Phaser crystallographic software. *J Appl Crystallogr* **40**, 658-674 (2007).

43. J. Huo *et al.*, Neutralization of SARS-CoV-2 by destruction of the prefusion Spike. *bioRxiv* 10.1101/2020.05.05.079202 (2020).

44. X. Chen *et al.*, Structural basis for antigen recognition by transglutaminase 2-specific autoantibodies in celiac disease. *J Biol Chem* **290**, 21365-21375 (2015).

45. P. Emsley, B. Lohkamp, W. G. Scott, K. Cowtan, Features and development of Coot. *Acta Crystallogr D Biol Crystallogr* **66**, 486-501 (2010).

46. P. D. Adams *et al.*, PHENIX: a comprehensive Python-based system for macromolecular structure solution. *Acta Crystallogr D Biol Crystallogr* **66**, 213-221 (2010).

47. N. C. Wu *et al.*, In vitro evolution of an influenza broadly neutralizing antibody is modulated by hemagglutinin receptor specificity. *Nat Commun* **8**, 15371 (2017).

48. D. Wrapp *et al.*, Cryo-EM structure of the 2019-nCoV spike in the prefusion conformation. *Science* **367**, 1260-1263 (2020).
